# Expanding the clinical and genetic spectrum of *RHO*-associated retinitis pigmentosa

**DOI:** 10.3389/ebm.2026.10893

**Published:** 2026-02-04

**Authors:** Rebeca A. S. Amaral, Olivia A. Zin, Rosane G. Resende, Debora N. Moraes, Mariana V. Salles, Gabriela D. Rodrigues, Fabiana L. Motta, Juliana M. F. Sallum

**Affiliations:** 1 Department of Ophthalmology and Visual Sciences, Universidade Federal de São Paulo, São Paulo, Brazil; 2 Instituto de Genética Ocular, São Paulo, Brazil; 3 Instituto Brasileiro de Oftalmologia (IBOL), Rio de Janeiro, Brazil; 4 Instituto de Olhos Carioca, Rio de Janeiro, Brazil; 5 Obras Sociais Irmã Dulce, Salvador, Brazil

**Keywords:** autosomal dominant, genotype-phenotype, retinal dystrophy, retinitis pigmentosa, rhodopsin

## Abstract

The majority of cases of autosomal-dominant retinitis pigmentosa (adRP) are associated with rhodopsin *(RHO)* variants. More than 290 pathogenic variants responsible for 25%–30% of adRP cases have been identified to date. This retrospective report focuses on *RHO* and RP cases in the Brazilian population. Patients with molecular confirmation of pathogenic variants in the *RHO* gene were included. Their clinical and genetic data were analyzed. Segregation analyses were included where possible. Cases were classified as generalized RP or sector RP according to fundus examinations and imaging data. The medical records of 43 patients from 34 families with *RHO*-associated RP were reviewed. Twenty-two disease-causing variants of the *RHO gene* and four previously unreported variants (c.317G>T; c.937-2A>T, c.272_283del, and c.530+1G>C) were identified. The majority of cases involved missense variants. The most prevalent variant was c.551A>G, p.(Gln184Arg), which was identified in seven patients (21%) from four families. One patient presented with the splice donor variant c.530+1G>C in the homozygous state, which was classified as pathogenic. Thirty-two patients presented with a generalized RP phenotype, and six patients were diagnosed with sector RP. This study provides information on the clinical and genetic features of *RHO*-associated RP in the Brazilian population, expanding the spectrum of *RHO* gene disease-causing variant frequencies.

## Impact statement

RHO is one of the most frequently implicated genes in autosomal-dominant Retinitis Pigmentosa, yet most existing data come from non-Latin American populations. By identifying and characterizing RHO variants and their allele frequencies in Brazilian patients, this work expands the international catalog of RHO mutations and refines genotype–phenotype correlations in a diverse genetic background. The discovery of novel and population-specific variants provides critical information for accurate genetic diagnosis, counseling, and variant interpretation in Brazil. This study enhances clinical and research capacities by improving molecular diagnosis, informing patient selection for gene-specific therapies, and contributing to equitable representation in global RP studies. Ultimately, these findings strengthen the foundation for precision medicine and future therapeutic advances in inherited retinal diseases.

## Introduction

Rhodopsin is a photopigment molecule and the most abundant protein in rod photoreceptors. It is primarily affected in retinitis pigmentosa (RP). By the late 1980s, rhodopsin was one of the best-understood visual proteins in terms of its structure, biochemistry, and genetics [1]. The rhodopsin gene *(RHO)* was the first gene for which RP-associated variants were identified [2]. Large families with autosomal-dominant RP (adRP) have been studied for linkages. The first link between RP and the *RHO* locus was reported in 1989, and mutations in *RHO* were identified in 1990 [3].

*RHO*-associated RP accounts for 20-30% of adRP cases [4], and approximately 4% of all RP [5] cases; more than 290 disease-causing RHO variants have been identified according to the ClinVar [6], UniProt [7], and Franklin Community Databases [8].

The most frequent phenotypes linked to *RHO*-associated RP are the generalized (classical) form and the sector form. While sector RP tends to progress more slowly than the generalized type, multiple studies have reported that it can ultimately develop into a generalized form [9, 10]. *RHO* variants have also been found in the autosomal-recessive (arRP) forms of RP [11].

Rhodopsin plays an essential role in the visual process, and even minor errors during gene transcription, translation, folding, processing, or transport to the correct cellular location can impair vision [12]. Previous studies have shown that the clinical features of *RHO*-associated RP correlate with specific protein domains affected by mutations [13].

This retrospective study explores the molecular mechanisms and phenotypic spectrum of *RHO*-associated RP in a Brazilian population.

## Materials and methods

This study was conducted in accordance with the Declaration of Helsinki, with strict protection of patient identity, and was approved by the Research Ethics Committee of the Universidade Federal de São Paulo (protocol number 5.113.810). Written informed consent was obtained where necessary to perform the molecular tests. During DNA sample collection for molecular testing, all the patients and/or their legal guardians provided written informed consent for the use of their personal medical data for scientific purposes and publication.

This observational retrospective study was performed. The inclusion criterion comprised genetically confirmed *RHO*-associated RP retrieved from the medical records of different ophthalmological centers in Brazil. Patient data from ophthalmological, genetic, clinical, and imaging records were evaluated. Genetic analysis was performed using commercial next-generation sequencing (NGS) panels for inherited retinal disorders, which included either 224 or 330 genes. Three of the most common genetic testing laboratories that were used were Invitae Laboratory, Mendelics, and Dasa Genomica. These genetic testing laboratories are accredited by the College of American Pathologists (CAP) and the Clinical Laboratory Improvement Amendments (CLIA). The pathogenicity of each variant was classified according to the American College of Medical Genetics and Genomics (ACMG) [14]. The *RHO* transcript ID is NM_000539.3. Two platforms combine computational predictions with clinical support, segregation, or functional studies to assist in variant classification. Both use sets of rules that follow the ACMG criteria: Franklin (https://franklin.genoox.com) and Varsome (https://varsome.com). Both were accessed on 25 October 2025. The identified variants were compared with records in ClinVar (https://www.ncbi.nlm.nih.gov/clinvar/; accessed on 25 October 2025). Segregation analyses were performed where available.

For all variants with sufficient evidence, the classification followed the system proposed by Athanasiou et al.: [15] Class 1: variants affecting post-Golgi trafficking and outer segment (OS) targeting; Class 2: variants involving misfolding, endoplasmic-reticulum (ER) retention, and protein instability; Class 3: variants disrupting vesicular trafficking and endocytosis; Class 4: variants altering post-translational modifications and reducing protein stability; Class 5: variants impairing transducin activation; Class 6: variants leading to constitutive receptor activation; and Class 7: variants resulting in dimerization deficiency.

## Results

Forty-three patients from 34 families with conclusive molecular genetic testing were identified as having *RHO*-associated RP. A total of 22 disease-causing variants of the *RHO* gene were classified as pathogenic or likely pathogenic. Four of these variants were previously unreported and were each identified in a different family (c.317G>T, c.937-2A>T, c.272_283del, and c.530+1G>C).

### Clinical characteristics

Six patients presented with a sector RP phenotype, and 32 patients presented with classical RP. One patient was an asymptomatic carrier and was evaluated for family history. Twenty-five patients had a positive family history (8 patients had an affected father, 8 patients had an affected mother, and 9 patients had an affected relative, such as a son, daughter, or cousin). The age at onset ranged from 5 to 38 years, with nyctalopia being the most common symptom. The best-corrected visual acuity (BCVA) ranged from 20/25 to 20/800. Eight patients presented with cystoid macular edema (CME) during the clinical course. The clinical characteristics are presented in Table 1.

**TABLE 1:** Clinical characteristics of RHO-associated RP patients.

Patients (*n* = 38)	Generalized RP (*n* = 32)	Sector RP (*n* = 6)
Families	26	5
Gender Male Female	12 (36.0%) 21 (63.0%)	3 (50.0%) 3 (50.0%)
Age of onset, mean (SD), years	18.1 (10.08)	27.5 (17.67)
First symptom	Nyctalopia (54.0%)	Nyctalopia (20.0%)
Baseline BCVA, mean (SD), LogMAR	0.43 (0.40) OD; 0.50 (0.50) OS	0.28 (0.31) OD; 0.08 (0.09) OS
Cystoid macular edema (CME)	7 (21.0%)	1 (20.0%)

### Molecular diagnosis

The majority of variants were missense (19 variants, 86.0%); the remainder included two splicing variants and one in-frame deletion. The most prevalent variant was c.551A>G, p.(Gln184Arg), which was identified in seven patients (21.0%) from four families. One patient presented with the homozygous splice donor variant c.530+1G>C, which was classified as pathogenic; subsequently, segregation analysis was conducted. Table 2 summarizes the variants and allele frequencies observed in this cohort (Supplementary Table S1).

**TABLE 2:** Pathogenic and likely pathogenic variants of *RHO*-associated RP patients.

Nucleotide change	Protein change	Allele frequency (families)	Variant type	GnomAD total allele freq (%)^a^	ACMG classification/criteria	First report
c.45T>G	p.(Asn15Lys)	1 (1)	Missense	-	Likely pathogenic/PS1, PM2, PM5, PP3	[16]
c.137T>G	p.(Leu46Arg)	3 (1)	Missense	-	Pathogenic/PS4, PM1, PM2, PP3	[17]
c.272_283del	p.(Thr92_Leu95del)	1 (1)	In-frame deletion	-	Likely pathogenic/PM1, PM2, PM4	This study
c.316G>A	p.(Gly106Arg)	4 (4)	Missense	0.000411	Pathogenic/PS1, PS3, PM1, PM2, PM5	[18]
c.317G>T	p.(Gly106Val)	4 (3)	Missense	-	Likely pathogenic/PM2, PM5, PP2, PP3, PP5	This study
c.341G>T	p.(Gly114Val)	1 (1)	Missense	-	Likely pathogenic/PM1, PM2, PM5, PP3, PP5	[19]
c.403C>T	p.(Arg135Trp)	5 (2)	Missense	0.000137	Likely pathogenic/PM1, PM2, PM5, PP3, PP5	[20]
c.404G>T	p.(Arg135Leu)	1 (1)	Missense	-	Likely pathogenic/PM1, PM2, PM5, PP3, PP5	[21]
c.491C>T	p.(Ala164Val)	1 (1)	Missense	0.0003979	Pathogenic/PS4, PM2, PM5, PM1, PP2, PP5	[15]
c.509C>G	p.(Pro170Arg)	1 (1)	Missense	0.0000684	Pathogenic/PS3, PM1, PM2, PM5, PP3	[18]
c.512C>A	p.(Pro171Gln)	1 (1)	Missense	-	Pathogenic/PS3, PM1, PM2, PM5, PP3	[18]
c.512C>T	p.(Pro171Leu)	1 (1)	Missense	-	Pathogenic/PS3, PM1, PM2, PM5, PP3	[18]
c.530+1G>C	(p.?)	2 (1)	Splicing	-	Pathogenic/PVS1, PM2, PP5	This study
c.533A>G	p.(Tyr178Cys)	1 (1)	Missense	0.0000684	Pathogenic/PS3, PM1, PM2, PM5, PP3	[22]
c.551A>G	p.(Gln184Arg)	7 (4)	Missense	0.000657	Likely pathogenic/PM1, PM2, PP2, PP3	[23]
c.557C>G	p.(Ser186Trp)	1 (1)	Missense	-	Likely pathogenic/PM1, PM2, PM5, PP2, PP3	[24]
c.560G>C	p.(Cys187Ser)	1 (1)	Missense	-	Likely pathogenic/PM1, PM2, PM5, PP2, PP3	[25]
c.568G>A	p.(Asp190Asn)	3 (3)	Missense	0.000137	Pathogenic/PS3, PM1, PM2, PM5, PP3	[26]
c.800C>T	p.(Pro267Leu)	1 (1)	Missense	0.0000684	Pathogenic/PS4, PM1, PM2, PM5, PP3,	[25]
c.937-2A>T	(p.?)	1 (1)	Splicing	-	Pathogenic/PVS1, PM2, PP5	This study
c.1033G>C	p.(Val345Leu)	2 (2)	Missense	0.0000684	Pathogenic/PS1, PM1, PM2, PM5	[15]
c.1040C>T	p.(Pro347Leu)	1 (1)	Missense	0.000137	Likely pathogenic/PM1, PM2, PM5, PP3, PP5	[27]

^a^ Accessed on December 2025.

### Variant class

**FIGURE 1:**
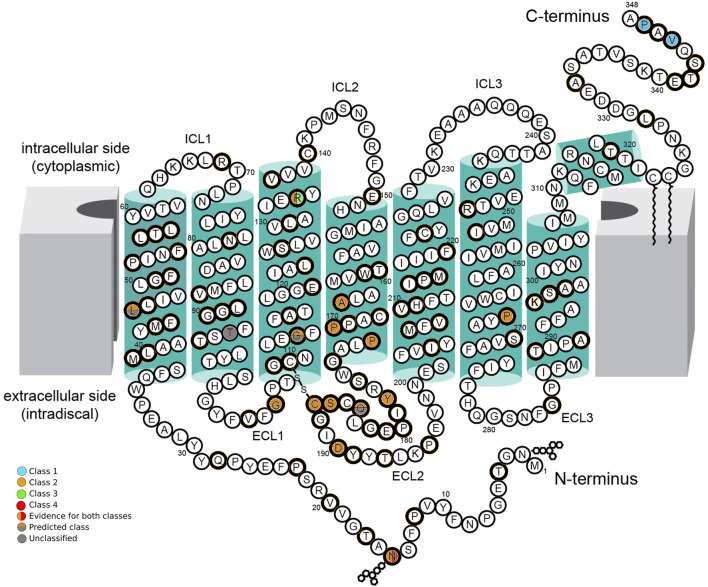
Schematic of the secondary structure of rhodopsin, Adapted from “Schematic rod photoreceptor and rhodopsin structure. (C) Two-dimensional representation of human Rho structure. Residues mutated in RP are indicated with orange circles. The Lys296, which covalently binds the 11-cis-retinal, is shown with a yellow circle filled with orange. The P23H mutation is shown with a red circle filled with orange” by Maria Azam and Beata Jastrzebska, licenced under CC BY 4.0. The seven-fold transmembrane helices, plus an eighth helix parallel to the membrane surface, are colored in green boxes. The intracellular side (cytoplasmic) contains three intracellular loops (ICL1, ICL2, and ICL3) and the carboxy-terminus (C-terminus) of the polypeptide chain. The extracellular side (intradiscal) contains the other three extracellular loops (ECL1, ECL2, and ECL3) and the amino-terminal end (N-terminus). The position of amino acid residues affected by *RHO* variants found in this cohort is indicated by colored circles. Class 1 variants (blue circles), Class 2 variants (orange circles), Class 3 variants (green circles), and Class 4 variants (red circles) are indicated with their location in the protein. Where there is evidence for more than one class type, it is shown with a vertical color split. Those with predicted effects are shown with a horizontal color split. Unclassified variants are indicated with gray circles.

Two variants were classified as Class 1, eleven were classified as Class 2, one variant as Class 2/3, one variant as Class 2/4, three variants as unclassified predicted Class 2, and three variants remained unclassified (U) due to a lack of experimental evidence (Figure 1; Table 3). Class 2 was the most prevalent in this cohort. Twelve Class 2 patients presented with a generalized RP phenotype, five patients had a sector RP phenotype, and three patients were unavailable for clinical classification. Two patients harbored Class 1 variants and presented with generalized RP. Three variants were unclassified but predicted to be Class 2; five patients presented with a generalized RP phenotype, and one was an asymptomatic carrier. One patient harbored a variant combining classes 2 and 4 (Class 2/4) with generalized RP. Five patients harbored variants combining Classes 2 and 3 (Class 2/3), and all exhibited a generalized RP phenotype.

**TABLE 3:** Variant class and phenotype correlation.

Nucleotide change	Protein change	Location	Suggested class	Phenotype (n)
c.45T>G	p.(Asn15Lys)	Intradiscal (N-terminal segment)	2/4	generalized RP (1)
c.137T>G	p.(Leu46Arg)	1st alpha helix (TM1)	U/P2	generalized RP (2)
c.272_283del	p.(Thr92_Leu95del)	2nd alpha helix (TM2)	U	sector RP (1)
c.316G>A	p.(Gly106Arg)	Intradiscal (1st extracellular loop)	2	generalized RP (2)/sector RP (2)
c.317G>T	p.(Gly106Val)	Intradiscal (1st extracellular loop)	2	generalized RP (2)/sector RP (2)
c.341G>T	p.(Gly114Val)	3rd alpha helix (TM3)	U/P2	generalized RP (1)
c.403C>T	p.(Arg135Trp)	3rd alpha helix (TM3)	2/3	generalized RP (5)
c.404G>T	p.(Arg135Leu)	3rd alpha helix (TM3)	3	generalized RP (1)
c.491C>T	p.(Ala164Val)	4th alpha helix (TM4)	2	N/A
c.509C>G	p.(Pro170Arg)	4th alpha helix (TM4)	2	N/A
c.512C>A	p.(Pro171Gln)	4th alpha helix (TM4)	2	generalized RP (1)
c.512C>T	p.(Pro171Leu)	4th alpha helix (TM4)	2	generalized RP (1)
c.530+1G>C	(p.?)	-	U	generalized RP (1)
c.533A>G	p.(Tyr178Cys)	Intradiscal (2nd extracellular loop)	2	generalized RP (1)
c.551A>G	p.(Gln184Arg)	Intradiscal (2nd extracellular loop)	U/P2	generalized RP (1)
c.557C>G	p.(Ser186Trp)	Intradiscal (2nd extracellular loop)	2	generalized RP (1)
c.560G>C	p.(Cys187Ser)	Intradiscal (2nd extracellular loop)	2	generalized RP (1)
c.568G>A	p.(Asp190Asn)	Intradiscal (2nd extracellular loop)	2	generalized RP (2)/sector RP (1)
c.800C>T	p.(Pro267Leu)	6th alpha helix (TM6)	2	generalized RP (1)
c.937-2A>T	(p.?)	-	U	generalized RP (1)
c.1033G>C	p.(Val345Leu)	Cytoplasm (C-terminal)	1	N/A
c.1040C>T	p.(Pro347Leu)	Cytoplasm (C-terminal)	1	generalized RP (1)

U, unclassified; P2, predicted class 2; N/A, not available.

### Retinal imaging

**FIGURE 2:**
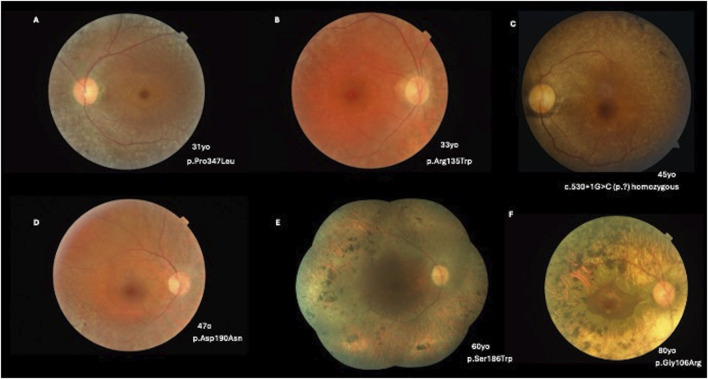
**(A)** A 31-year-old patient with a c.1040C>T, p.(Pro347Leu) variant presenting with BCVA of 20/25 OD and 20/30 OS, pigmentary mottling, and peripheral chorioretinal atrophy with bone-spicule hyperpigmentation. **(B)** A 33-year-old patient with a c.403C>T, p.(Arg135Trp) variant (BCVA: 20/40 in both eyes) with peripapillary and peripheral chorioretinal atrophy with narrowed vessels. **(C)** A 45-year-old patient with c.530+1G>C (p.?) in homozygosity (BCVA: 20/80 OD; 20/100 OS) and a more severe phenotype of classical RP. **(D)** A 47-year-old patient with a c.568G>A, p.(Asp190Asn) variant (BCVA: 20/40 in both eyes) with pigmentary mottling and peripheral chorioretinal atrophy. **(E)** A 60-year-old patient with a c.557C>G, p.(Ser186Trp) variant (BCVA: 20/400 in both eyes) with diffuse pigmentary bone-spicules and peripheral chorioretinal atrophy. **(F)** An 80-year-old patient with a c.316G>A, p.(Gly106Arg) variant (BCVA: 20/100 in both eyes) with advanced classical RP findings and preserved central vision in the macular area.

**FIGURE 3:**
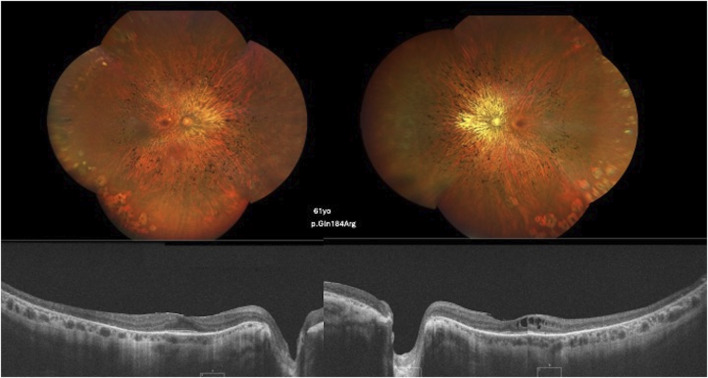
Color fundus and SD-OCT image of a 61-year-old patient carrying the c.551A>G, p.(Gln184Arg) variant showing diffuse classical RP findings and atrophy of the retinal layers, with the ellipsoid zone relatively preserved in the foveal area. CME is observed in the left eye.

Thirty-two patients exhibited a generalized RP phenotype. Color fundus photography revealed common findings, including bone-spicule pigment deposits, a mottled retinal fundus, and vessel attenuation. Among these patients, seven presented with macular edema on optical coherence tomography (OCT) scans. Figures 2, 3 illustrate fundus images of *RHO*-associated RP patients in this study.

**FIGURE 4:**
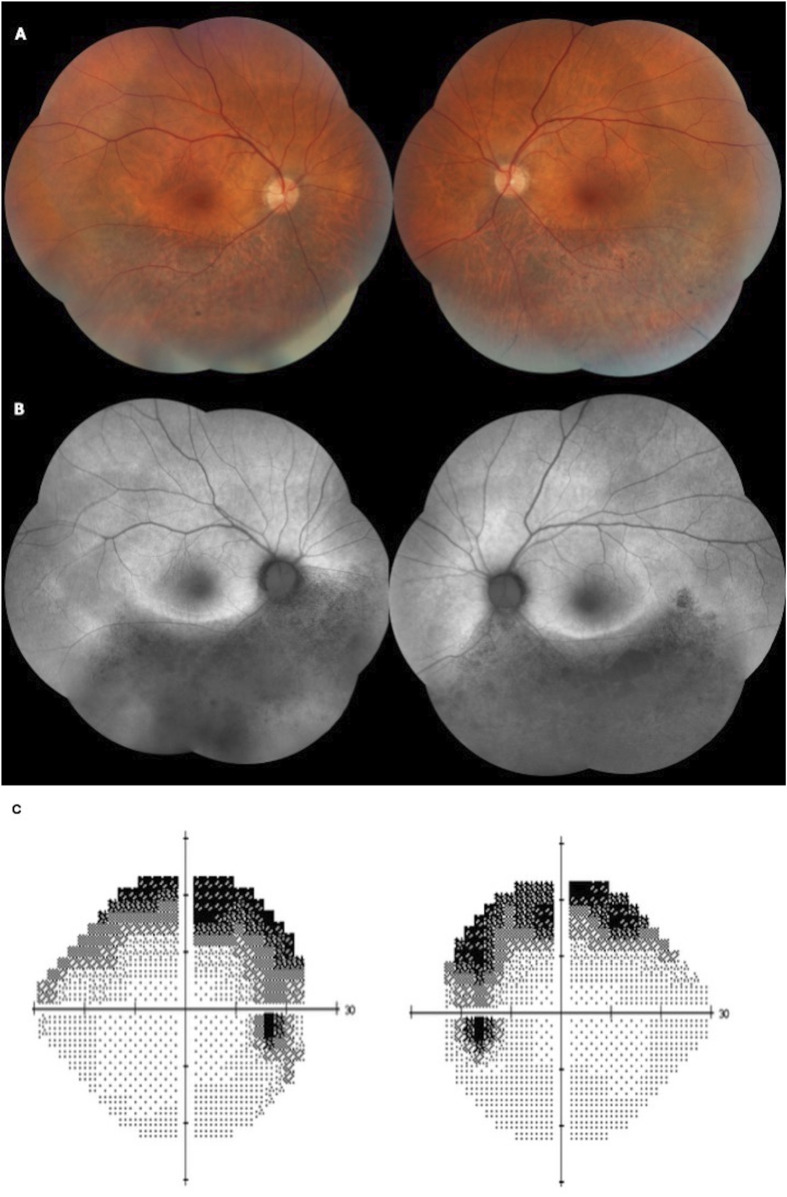
Color fundus **(A)** and fundus autofluorescence (FAF) **(B)** of a 58-year-old patient presenting with BCVA of 20/25 in both eyes and sectoral inferior RP. The patient has the heterozygous variant c.568G>A, p.(Asp190Asn). **(C)** A Humphrey 24–2 grayscale visual field map of the same patient with bilateral and symmetrical superior visual field defects, showing anatomo-functional correlation with the fundus images.

Six patients presented with the sector RP phenotype. The retinal fundus typically exhibited bone-spicule pigment deposits in the inferior retina. One patient presented with macular edema. Figure 4 presents the findings for sector RP.

## Discussion

*RHO*-associated RP is one of the most common and well-characterized forms of adRP [28, 29]. Clinically, *RHO*-associated RP can present with distinct phenotypic patterns, ranging from diffuse retinal degeneration with early night blindness and peripheral vision loss to sector RP, in which degeneration is confined to specific retinal regions and disease progression is slower [30].

This study describes the first Brazilian cohort with *RHO*-associated RP, and the clinical and molecular spectrum related to retinal degeneration.

Approximately 60.0% of patients presented with a family history of RP. In total, 85% of patients had generalized RP, the most prevalent phenotype. Five patients had sector RP affecting the inferior retina, which is the most commonly affected retinal region. One hypothesis is that light exposure, particularly in the lower retinal regions that receive more direct illumination, contributes to disease progression [30]. In support of this hypothesis, studies using animal models of *RHO*-associated RP have shown that complete light deprivation can reduce the extent of outer retinal degeneration [31, 32].

Rhodopsin is a visual receptor composed of seven transmembrane helices connected by three extracellular loops on the intradiscal side and three intracellular loops on the cytoplasmic side [24]. Misfolding and ER retention are the most prevalent pathogenic mechanisms (Class 2) [33]. Class 2 variants were the most prevalent, with both generalized (63.0%) and sector (26.0%) RP phenotypes.

Several *RHO*-associated variants are responsible for sector RP; these are exclusively missense mutations, predominantly located in the intradiscal domain [30, 34]. Accordingly, the majority of patients in this cohort harbored intradiscal-domain missense variants. The exception was a sector RP patient with a previously unreported deletion variant in the second alpha-helix (TM2). The c.316G>A, p.(Gly106Arg) and c.568G>A, p.(Asp190Asn) variants, both frequently described as sector RP [10, 34], were identified in patients presenting with generalized RP. Similarly, the variant c.317G>T, p.(Gly106Val) was identified in two patients with sector RP and two with generalized RP. This is a previously catalogued variant without published clinical correlation (dbSNP rs1578278417). This missense variant is also a Class 2 variant, located intradiscally in the first extracellular loop and affecting codon 106. In this analysis, no variant was exclusive to the sector RP cases.

Cytoplasmic-domain variants are typically associated with a severe RP phenotype, characterized by the early rod and cone photoreceptor degeneration. In contrast, mutations affecting the extracellular domain are generally linked to a milder clinical presentation, with relatively preserved photoreceptor function and a slower rate of disease progression [35]. Class 1 variants in this cohort presented a mild phenotype, generalized RP, and early onset of symptoms. Class 2 variants are the most common, demonstrating a broader spectrum of clinical severity. Class 3 variants demonstrate early disease onset and a more severe phenotype. Variants in the N-terminal segment are sometimes associated with a relatively mild disease course, with RP developing later in life and slowly advancing symptoms [15]. In contrast, the patient described here with this variant location presented with generalized RP, high myopia, and early-onset symptoms, with relatively preserved vision until the sixth decade of life.

In this study, the c. 551A > G, p. (Gln184Arg) variant was the most frequent variant, found in seven patients from four families. The second most common variant was c.403C>T, p.(Arg135Trp), which was identified in five patients from two families. These two variants are present in European, American, and Asian populations. This is consistent with the literature, as missense mutations are the most common type of variant in the *RHO* gene [36].

*RHO* is one of the few genes that cause both adRPs and arRPs. The recessive form is typically associated with a complete loss of rhodopsin function, whereas the dominant form results from a gain-of-function and/or a dominant-negative mechanism [15]. To date, eight homozygous variants have been described in the *RHO* gene: c.448G>A, p.(Glu150Lys [37]; c.759G>T, p.(Met253Ile) [38]; c.931A>G, p.(Lys311Glu) [39]; c.482G>A, p.(Trp161*) [40]; c.745G>T, p.(Glu249*) [41]; c.936+1G>T (p.?) [42]; c.408C>A, p.(Tyr136*) [43]; and c.82C>T, p.(Gln28*) [23].

The underlying mechanisms by which missense mutations cause the recessively inherited form remain unclear; it is possible that missense changes are mild mutations that only become pathogenic when present on both alleles.

Aberrant splicing frequently generates premature termination codons (PTCs), which can result in the production of truncated proteins [44]. However, PTCs can trigger nonsense-mediated mRNA decay (NMD), an essential mRNA quality-control mechanism that clears flawed transcripts. Typically, mRNA transcripts are targeted for accelerated degradation by NMD when a PTC is located 50–55 nucleotides downstream of the final exon-exon junction [45]. This process prevents the translation of transcripts into potentially harmful truncated proteins, although the efficiency of this process is currently unknown.

Hernan et al. described that the adRP-causing *RHO* variant c.937-1G>T abolishes the canonical splice-acceptor site in intron 4 [46]. Consequently, an aberrant exonic splice-site was used during transcription, leading to the production of a protein lacking 13 amino acids. In contrast, the c.936+1G>T variant, located at the donor site of the same intron, results in the complete skipping of exon 4 and causes the recessive form of the disease.

In our cohort, we identified the c.937-2A > T variant, affecting the splice-acceptor site of intron 4. This is a novel allele at a known pathogenic site (dbSNP rs1578281565). Similar to the previously reported c.937-1G>T variant, the c.937-2A>T variant causes adRP with a severe generalized phenotype. Notably, the transcript resulting from this variant is predicted to evade NMD. Since the variant is located in the final intron, any resulting PTC would lie downstream of the final exon-exon junction, thus failing to meet the canonical ∼50 nt rule for NMD targeting. Although the exact consequences require functional studies, this NMD evasion suggests the production of a truncated protein.

In the context of homozygous *RHO* variants, NMD activation may lead to a marked reduction or complete absence of rhodopsin mRNA, resulting in functional null alleles [47]. Retinal degeneration in these cases may arise from the loss of rhodopsin expression rather than from the dominant-negative or gain-of-function effects typically associated with certain heterozygous *RHO* cases [47].

Another previously reported splicing variant is c.531-2A>G [46, 48]. Due to its intron 2 location, this variant was initially anticipated to undergo NMD and, consequently, manifest as arRP. However, this specific allele has been documented in the Spanish population, where it is linked to full adRP penetrance [48]. In support of this dominant mechanism, *in vitro* studies conducted by Hernan et al. demonstrated that the transcripts generated as a consequence of the c.531-2A>G variant were not entirely abolished by NMD. Consequently, a truncated protein is expressed, representing the probable cause of the adRP phenotype [46].

In this Brazilian cohort, a previously unreported variant was located in intron 2 and affected the splice donor site. The homozygous c.530+1G>T variant was detected in one patient diagnosed with RP at 25 years of age. The patient presented with early-onset symptoms, including nyctalopia, starting at 5 years of age. Unlike the c.531-2A>G variant, the c.530+1G>T variant appeared to be completely targeted by NMD. This hypothesis is supported by the inheritance pattern: only patient who possesses both affected alleles (homozygous) presents with the phenotype, whereas patients who carry a single heterozygous variant, such as this specific patient’s mother, remains asymptomatic.

The c.1040C>T, p.(Pro347Leu) variant is the most frequently observed causative variant worldwide. It has also been identified in other ethnic groups [49]. In this Brazilian cohort, only one patient was identified with this variant, with generalized RP and a mild symptom phenotype.

*RHO* c.68C>A, p.(Pro23His) was the first variant reported at high frequency for this gene in the United States [2]. Based on a meta-analysis of diagnosed cases reported in the literature, the estimated clinical prevalence of adRP due to *RHO* c.68C>A, p (Pro23His) is approximately 2,000–3,000 patients [50]. In comparison, the number of individuals heterozygous for this variant in the United States was 6,176 [50].

Several techniques have been explored to treat *RHO*-associated retinopathy, many of which involve the c.68C>A, p.(Pro23His) variant [51–53], which has been comprehensively elucidated at the molecular level, with robust animal models available and a high potential clinical impact in the U.S. population.

However, the frequency of this variant is low in other populations. It appears to be extremely rare or even absent in populations outside the United States, with apparent geographical restrictions on this variant. A study of 300 Chinese families with RP found that, while *RHO* variants accounted for approximately 2.7% of cases, the c.68C>A, p.(Pro23His) variant was not reported in that population or in other Asian ethnic groups [54], such as Korean [55] and Japanese [56] cohorts, and only one case was reported in a large European cohort [57]. However, this was not observed in the Brazilian cohort.

This study has some limitations. One major limitation is the lack of functional assays to directly evaluate the molecular consequences of the identified *RHO* variants. Without experimental validation such as RNA expression analyses, minigene splicing assays, or protein quantification, it is impossible to conclusively determine whether the observed variants lead to RNA decay, aberrant splicing, or residual protein production. Functional investigations are imperative to confirm the molecular consequences of these variants and to clarify their contribution to phenotypic variability.

The genotype–phenotype correlations observed in this study should be interpreted as descriptive rather than causal or definitive associations, given the observational nature of the data and the limited sample size. Further genetic analyses of larger cohorts are required to better understand their pathophysiology.

In conclusion, this study provides valuable insights into the clinical and genetic characteristics of *RHO*-associated RP within the Brazilian population while broadening the documented spectrum of disease-causing *RHO* gene variants.

## Data Availability

The datasets presented in this study can be found in online repositories. The names of the repository/repositories and accession number(s) can be found in the article/Supplementary Material.
